# Complementary use of statistical parametric mapping and gait profile score to describe walking alterations in multiple sclerosis: a cross-sectional study

**DOI:** 10.1038/s41598-023-36916-5

**Published:** 2023-06-28

**Authors:** Fabiola Giovanna Mestanza Mattos, Francesco Luciano, Tiziana Lencioni, Elisa Gervasoni, Johanna Jonsdottir, Denise Anastasi, Gaspare Pavei, Mario Clerici, Davide Cattaneo

**Affiliations:** 1 Department of Pathophysiology and Transplantation, Università Degli Studi di Milano, 20100 Milan, Italy; 2 IRCCS Fondazione Don Carlo Gnocchi, Via Capecelatro 66, 20148 Milan, Italy

**Keywords:** Multiple sclerosis, Rehabilitation, Biomedical engineering

## Abstract

Gait analysis is often used to study locomotor alterations in people with multiple sclerosis (PwMS), but the large number of extracted variables challenges the interpretability. In this paper, we analysed gait alterations by combining the Gait Profile Score (GPS), which summarizes kinematic locomotor deviations, and Statistical Parametric Mapping (SPM), which compares kinematics and kinetics over the whole gait cycle. Eleven PwMS and 11 speed-matched Healthy Controls (HC) underwent overground gait analysis. GPS were compared through independent-samples t-tests; sagittal-plane kinematics and power at hip, knee, and ankle were compared through SPM Hotelling’s-T2 and SPM t-tests. Spearman’s correlation coefficients (r) between GPS and clinical outcomes were also calculated. PwMS had higher GPS than HC (PwMS = 8.74 ± 2.13°; HC = 5.01 ± 1.41°;*p* < 0.001). Multivariate SPM found statistically significant differences at 0–49%, 70–80%, and 93–99% of stride (*p* < 0.05) and univariate analysis showed reduced ankle dorsiflexion, and lower knee flexion during pre-swing and swing. GPS correlated with Expanded Disability Status Scale (r = 0.65; 95%C.I.[0.04,0.91]; *p* = 0.04) and 2-Minute Walking Test (r = -0.65; 95%C.I.[-0.91,-0.04]; *p* = 0.04). GPS in conjunction with SPM revealed multi-joint kinematic alterations on sagittal plane involving distal joint angles, ankle and knee, during the stance phase with no changes at the proximal level. Gait deviations were more pronounced in PwMS with higher disability and walking limitations.

## Introduction

Multiple sclerosis is the most common demyelinating disease and one of the main causes of non-traumatic disability in young adults^1^ affecting 2.0–2.4 million people worldwide^2^ Even if its clinical manifestations are heterogeneous both in symptoms and progression, 85% of people with multiple sclerosis (PwMS) present mobility limitations and perceive gait disturbances as their most prevalent source of disability^3,4^

Such gait disturbances have been investigated by acquiring spatiotemporal, kinematic, and kinetic metrics with three-dimensional motion capture systems^5^ When compared with healthy participants, PwMS present lower preferred walking speed, reduced stride and step length, and cadence with increased double support time and gait asymmetry^4,6^ These parameters can also discriminate between different disability levels^7^.

Integrating information from kinematic and kinetic measurements allow for a thorough evaluation of gait in PwMS; however, kinematic changes in this population typically affect more joints and the dimensionality of such data poses some challenges limiting their clinical interpretation. These limitations can be overcome by using scalar summary measures such as the Gait Profile Score (GPS), an index which quantifies gait quality in PwMS based on the deviation of multiple joint kinematics from a normal physiological reference^8,9^The GPS can also be deconstructed into a movement analysis profile composed of 9 Gait Variable Scores (GVS), providing insight into which kinematic variables contribute to the increase in the summary index^9^ Therefore, GPS represents a useful and immediate tool to support clinical practices. In a recent study, Pau et al. found increased hip flexion and reduced knee flexion–extension as well as reduced plantar flexion along with reduced joint torque and power at hip, knee, and ankle in the sagittal plane in PwMS. They also showed that these alterations increased with disability, measured by Expanded Disability Status Score (EDSS)^10^ Nonetheless, as each angular measurement is a time-dependent waveform, summary measures like GPS fail to detect specific phases of gait cycle in which statistically significant alterations occur.

This drawback can be overcome by comparing kinematic and kinetic waveforms through Statistical Parametric Mapping (SPM)^11^ This method allows for hypothesis tests over whole waveforms instead of single, discrete points or summary measures, identifying statistically significant differences across the phases of the gait cycle. Another key advantage of SPM is the possibility to perform univariate and multivariate analyses of multiple joint angles, and therefore to consider both single joints characteristics and the interactions between the curves simultaneously^12^.

To date, studies on walking kinematics and kinetics in PwMS have been conducted using either discrete point analysis or summary measures; hence evidence is lacking on time-dependent features of gait which could be unveiled by SPM, and how coherent summary measures and SPM analyses are. Therefore, this cross-sectional study aims at identifying gait alterations in PwMS with moderate to severe disability, quantifying deviations from normal physiological patterns using GPS and SPM analysis on hip, knee, and ankle kinematics. In addition, we provided exploratory analyses on kinetics to better clarify results from the kinematics.

In accordance with the previous study by Pau, we hypothesized that GPS will be higher in PwMS compared to Healthy Controls (HC) and that GVS of PwMS will be more affected on sagittal plane compared to horizontal and frontal planes^10^ Therefore, we restricted our primary analyses to sagittal plane kinematics without *a-priori* hypothesis on the most affected joints or phases of gait cycle.

## Methods

### Study design

Data for this cross-sectional study were collected from a randomized control trial (ClinicalTrials.gov Identifier: NCT03271125), about motor rehabilitation in PwMS recruited in Don Gnocchi Foundation Milan, Italy. The study was approved by the Ethical Committee of IRCCS Don Carlo Gnocchi Foundation, (Milan, Italy) and was carried out in accordance with the principles of Declaration of Helsinki. Written informed consent was obtained from all participants.

### Participants

According to sample size estimation (see Supplementary material), 11 PwMS with relapsing–remitting, primary progressive and secondary progressive MS according to the 2005 Mc Donald criteria were included in this study (see Table 1)^13^ Inclusion criteria were age > 18 years, Expanded Disability Status Scale (EDSS) score ≤ 6.5^14^ stable neurological condition (no relapsed and steroid-free for at least 1 month prior to the evaluation), and capacity to understand and follow instructions. Excluded were participants presenting any musculoskeletal and/or other neurological disorders that could influence gait and balance. A control group of 11 HC matched for sex, age, anthropometric measures, and speed provided kinematic and kinetic gait reference data; included participants exhibited a normal joint range of motion and muscle strength, without any gait or balance deficit.

**Table 1:** Demographic and clinical characteristics of PwMS and HC.

	PwMS	HC	*p* value
	(n = 11)	(n = 11)	
Female (n)	5 (46%)	7 (64%)	0.69†
Age (years)	51 (11.5)	38.9 (17.6)	0.10*
Height (m)	1.70 (0.10)	1.70 (0.09)	0.97*
Body mass (kg)	68.8 (13.2)	65.7 (15.6)	0.62*
Walking speed (m/s)	0.60 (0.27)	0.58 (0.1)	0.85*
Walking speed norm (%BH/s)	35.2 (14.6)	34.2 (4.8)	0.84*
Type of MS (n, %)			
RR	7.64%	–	–
PP	2.18%		
SP	2.18%		
EDSS (points)	5.5 (0.7)	–	–
Onset (years)	16.6 (5.8)	–	–
2MWT (m)	88.9 (30.8)	–	–
DGI (points)	13.1 (4.9)	–	–
BBS (points)	41.1 (10.6)	–	–

Data are reported as mean (SD) or number (%). *PwMS* People with multiple sclerosis, *HC* Healthy controls, *PP* Primary progressive, *SP* Secondary progressive, *RR* Relapsing remitting, *EDSS* Expanded disability status scale, *2MWT* Two minute walk test, *DGI* Dynamic gait index, *BBS* Berg balance scale. *p* value * two-tailed, independent-samples t-test, †chi-square test.

### Clinical outcomes

Disability was quantified by the EDSS score^14^ Gait endurance was measured by the 2-min walk test (2MWT)^15^, while the static and dynamic balance were measured through the Berg Balance Scale (BBS)^16^ and the dynamic gait index (DGI)^17^.

The most affected side of PwMS was identified according to items 13 and 14 of the BBS. Item 14 was used to assess single leg except for three participants that were not able to perform item 14 with both legs. In this case we used item 13 in which the subject stood unsupported with one foot in front of the other testing the posterior foot performance. The procedure was repeated for both sides, and the most affected side was the one with the worst performance^18^.

### Kinematic and kinetic data collection

Kinematic and kinetic data were collected both from PwMS and HC walking in a 10-m corridor. Following the total-body LAMB marker set, 29 retro-reflective markers were placed on the head, upper limbs, trunk, pelvis, and lower limbs; a 9-camera SMART-D optoelectronic system (BTS, Milano, Italy) was located around a 4 m × 2 m × 2 m acquisition volume (sampling at 200 Hz)^19^ Finally, a force plate (Kistler, Winterthur, Switzerland) recorded ground reaction forces (sampling at 960 Hz), needed to compute kinetic data. Motion capture and ground reaction forces data were automatically synchronized.

All the participants performed at least 5 gait trials at their self-selected speed^20^ in addition, HC were asked to perform trials at slower speeds to provide a reference dataset that was speed-matched with PwMS.

### Kinematic and kinetic data elaboration

After the acquisition, data processing was performed using Matlab (version R2022a, Math-Works, Natick, MA, USA). The markers’ coordinates were low-pass filtered at a cut-off frequency of 6 Hz, and anthropometric parameters were computed from markers’ positions recorded during the calibration trial according to the LAMB protocol to estimate the internal joint centres^19^ Gait events were detected through manual annotation based on video data.

Finally, mass-normalized powers at hip, knee, and ankle joints were computed using inverse dynamics; joint angles and powers time series were normalized in 101 time points over one stride. In locomotion biomechanics, mass normalization allows the minimization of changes due to body mass, emphasizing differences in joint force production between subjects with different anthropometric characteristics. Since the gait alterations are generally asymmetric in PwMS, the most impaired lower limb was investigated.

### Gait profile score (GPS)

The GPS is a summary measure of gait quality. It is defined as the root mean square difference (expressed in degrees) between kinematic data from PwMS and the mean value of the HC group. Such difference is calculated along the whole gait cycle over nine kinematic variables: pelvic tilt, rotation and obliquity, hip flexion–extension, adduction–abduction and rotation, knee flexion–extension, ankle dorsiflexion, and foot progression, further details are provided in the Supplementary material. The average root mean square difference for each of the nine kinematic variables is named Gait Variable Score (GVS); thus, GPS is the root mean square of the nine Gait Variable Scores^9^ Higher values of GVS and GPS indicate a greater difference of PwMS’s kinematics from normal physiological reference gait^21^.

### Statistical parametric mapping

#### Sample size estimation

A priori SPM power analysis was conducted to calculate the required sample size for kinematic comparisons, which was the primary endpoint of this study^22–24^ Eleven participants for each group were hence recruited in order to keep the false-negative and false-positive error rates below 0.20 and 0.05, respectively (Supplementary Fig. 1); analyses were conducted using Python 3.9.7, Numpy 1.21.2, and Power1D 0.1.4^24–26^ Further information on SPM and power analysis is provided in the Supplementary material.

#### Hypothesis testing

Joint angles time series were compared between PwMS and HC through multivariate SPM Hotelling’s T^2^ test, followed by univariate SPM t-test comparisons for each joint. Finally, non-frontal plane joint kinematics and joint power time series were investigated in an explorative analysis to further explain kinematic results. For each test, the SPM{T^2^} or SPM{t} statistics were calculated, together with the null hypothesis rejection threshold with a false-positive error rate of 0.05. The family-wise error rate of the SPM t-tests was kept below 0.05 through the Holm-Bonferroni procedure. Comparisons were considered statistically significant if the test statistics exceeded the rejection threshold in one or more continuum points. In this case, the points above the threshold formed one or more clusters, whose *p* values were calculated and reported. The analysis was done using Python 3.9.7, Numpy, and spm1d^24–26^. The relationship between GPS and clinical outcomes (EDSS, 2MWT, DGI, and BBS) was assessed by calculating Spearman’s correlation coefficient with the level of significance of 0.05. Analyses were conducted using R (version 4.1.0)^21^.

## Results

### Demographic data

Demographic characteristics of the participants and clinical outcomes are shown in Table 1, while Table 2 shows the GPS and GVS values for HC and the most impaired leg of PwMS (sorted by t-value). With respect to the sagittal plane, larger statically significant differences (two-tailed, independent-samples t-test) were observed for distal segments (i.e., knee and ankle), while those related to the hip and pelvis kinematics were smaller.

**Table 2:** Comparison between GPS and GVS values in PwMS versus HC.

	GPS and GVS scores	PwMS	HC	Differences PwMS vs HC		
	Degrees	Mean (SD)	Mean (SD)	$\Delta$	*t* value	*p* value
	GPS	8.74 (2.13)	5.01 (1.41)	3.73	4.84	< 0.001
	Pelvic obliquity	3.64 (0.93)	1.57 (0.59)	2.07	6.25	< 0.001
	Knee flexion–extension	12.82 (3.40)	5.00 (2.57)	7.82	6.08	< 0.001
	Ankle dorsi- and plantar-flexion	9.28 (3.82)	4.26 (1.64)	5.02	4.00	0.001
	Hip abduction–adduction	5.00 (2.25)	2.12 (1.12)	2.8 8	3.81	0.002
GVS	Pelvic rotation	6.02 (2.32)	3.45 (1.87)	2.57	2.87	0.01
	Foot progression	8.30 (4.31)	4.55 (2.55)	3.75	2.48	0.02
	Hip flexion–extension	8.21 (4.08)	5.48 (2.04)	2.73	1.98	0.07
	Pelvic tilt	6.81 (3.66)	4.11 (3.35)	2.70	1.81	0.09
	Hip rotation	9.78 (5.83)	7.39 (5.27)	2.39	1.01	0.32

*GPS* Gait profile score, *GVS* Gait variability score, *PwMS* People with multiple sclerosis, *HC* Healthy controls. Data are reported as mean (SD). t statistics and *p* values from two-tailed, independent-samples t-test are reported.

### Multivariate analysis of kinematic data

A statistically significant difference (*p* < 0.001; independent-samples t-test) in GPS was found between PwMS (GPS = 8.74 ± 2.13°) and HC (GPS = 5.01 ± 1.41°). A reduced GPS version considering only the sagittal angles of the three joints was also calculated, and the difference between the two groups was more pronounced (PwMS = 10.57 ± 2.79°; HC = 5.18 ± 1.36°; *p* < 0.001; independent-samples t-test).

**Figure 1:**
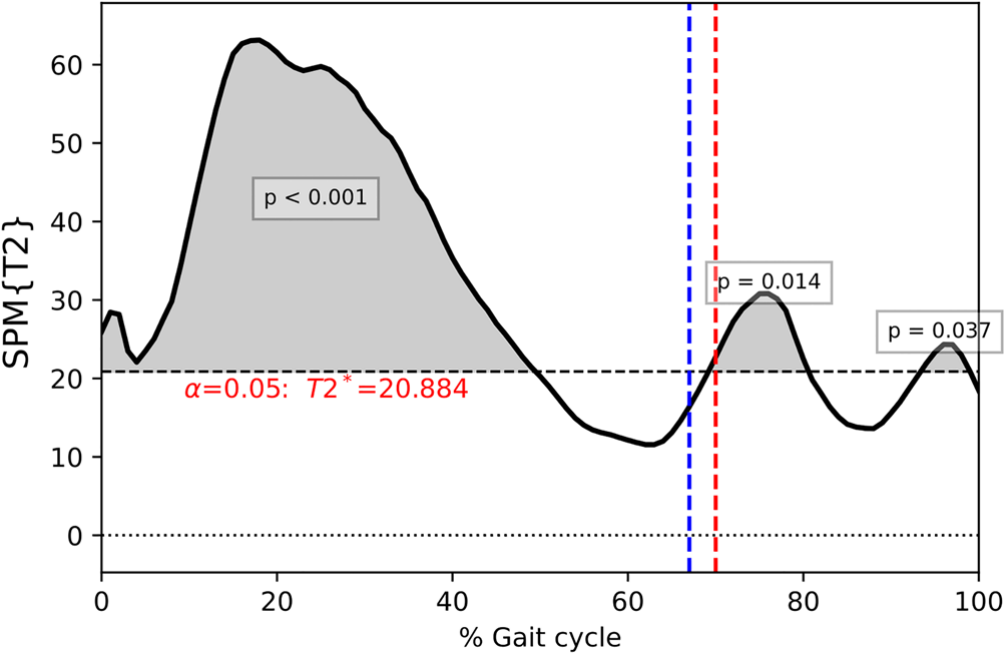
SPM Hotelling’s T2 test comparing hip, knee, and ankle sagittal angles of PwMS and HC. Multivariate SPM Hotelling’s T^2^ test. *SPM* Statistical parametric mapping; *PwMS* People with multiple sclerosis; *HC* Healthy controls; Horizontal black dashed line, Critical threshold for the SPM Hotelling’s T^2^ test; grey area, supra-threshold cluster; vertical dashed lines, mean foot-off in PwMS (red) and HC (blue).

Coherently, when comparing the three-joint kinematics between HC and PwMS through Hotelling’s SPM test, the T^2^ statistics exceeded the null hypothesis rejection threshold (Fig. 1). Most of the alterations in PwMS gait kinematics occurred during the stance phase (0–49% of gait cycle), followed by two clusters in the swing phase (70–80% of gait cycle) and terminal swing, immediately before the next initial contact (93–99% of gait cycle).

### Univariate analysis of kinematic data

#### Ankle joint

**Figure 2:**
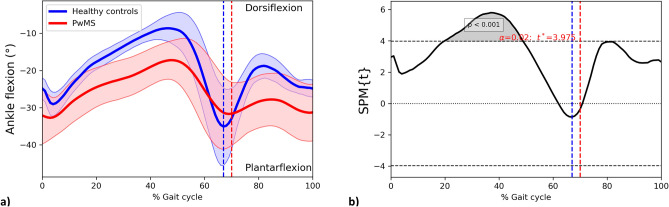
The ankle dorsi-and plantar flexion angle curves for PwMS and HC throughout the gait cycle. (**a**) Mean trajectories for ankle dorsi- and plantar flexion angles in PwMS (red) and HC (blue), Standard Deviations of trajectories for ankle dorsi- and plantar flexion angles in PwMS (shaded red area) and HC (shaded blue area). (**b**) Results of independent-samples SPM t-test. *PwMS* People with multiple sclerosis; *HC* Healthy controls; *SPM* Statistical parametric mapping; Horizontal black dashed line, critical threshold for the two-sample SPM t-test; grey area, supra-threshold cluster; vertical dashed lines, mean foot-off in PwMS (red) and HC (blue).

Alterations observed in the multivariate SPM analysis were mainly due to abnormal ankle and knee joint kinematics. The ankle joint GVS was significantly different in PwMS compared with HC (Ankle dorsi- and plantar-flexion GVS PwMS = 9.28 ± 3.82°; HC = 4.26 ± 1.64°; *p* = 0.001; independent-samples t-test) and SPM analysis revealed a cluster of statistically significant reduction in dorsiflexion between 20–49% of gait cycle (*p* < 0.001, two-sample SPM t-test; Fig. 2).

#### Knee joint

**Figure 3:**
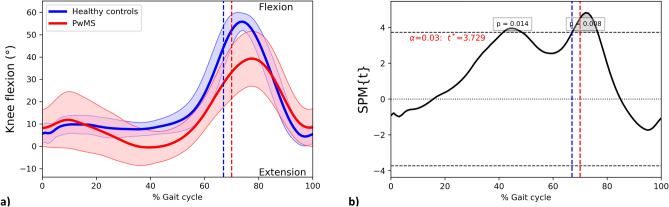
The knee flexion–extension angle curves for PwMS and HC throughout the gait cycle. (**a**) Mean trajectories for knee flexion–extension angles in PwMS (red) and HC (blue), Standard Deviations of trajectories for ankle dorsi- and plantar flexion angles in PwMS (shaded red area) and HC (shaded blue area). (**b**) Results of the independent-samples SPM t-test. *PwMS* People with multiple sclerosis; *HC* Healthy controls; *SPM* Statistical parametric mapping; Horizontal black dashed line, Critical threshold for the two-sample SPM t-test; grey area, supra-threshold cluster; vertical dashed lines, mean foot-off in PwMS (red) and HC (blue).

A large between-group difference in GVS was observed for knee joint kinematics on the sagittal plane (knee flexion–extension GVS PwMS = 12.82 ± 3.40°; HC = 5.00 ± 2.57°; *p* < 0.001; independent-samples t-test) with a statistically significant reduction in flexion from 44 to 45% and from 68 to 76% of gait cycle (two-sample SPM t-test; Fig. 3). The p-values for these supra‐threshold clusters were 0.014 and 0.008, respectively.

#### Hip joint

**Figure 4:**
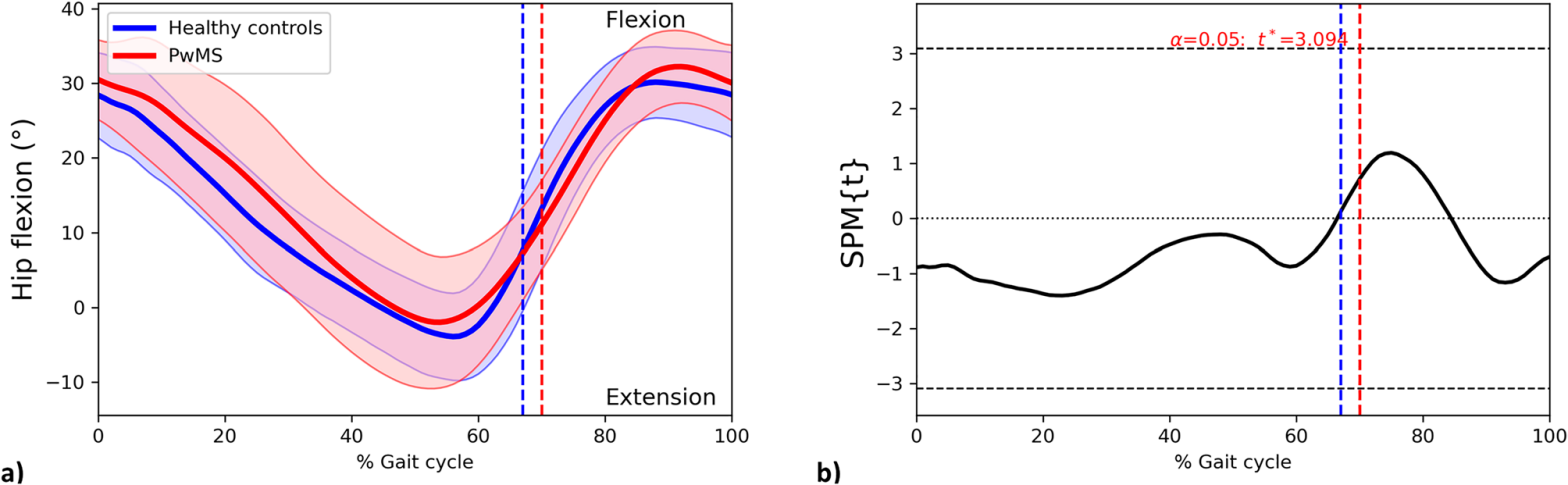
The hip flexion–extension angle curves for PwMS and HC throughout the gait cycle. (**a**) Mean trajectories for hip flexion–extension angles in PwMS (red) and HC (blue) walking, Standard Deviations of trajectories for ankle dorsi- and plantar flexion angles in PwMS (shaded red area) and HC (shaded blue area). (**b**) Results of the independent-samples SPM t-test. *PwMS* People with multiple sclerosis; *HC* Healthy controls; *SPM* Statistical parametric mapping; Horizontal black dashed line, critical threshold for the two-sample SPM t-test; vertical dashed lines, mean foot-off in PwMS (red) and HC (blue).

Sagittal-plane hip kinematics did not differ between PwMS and HC, as evidenced by the non-significant differences in GVS (Hip Flexion–extension GVS PwMS = 8.21 ± 4.08°; HC = 5.48 ± 2.04°; *p* = 0.07; independent-samples t-test) and SPM comparisons (Fig. 4). Hip abduction–adduction and pelvic obliquity GVS differed between groups (Hip Abduction–adduction GVS PwMS = 5.00 ± 2.25°; HC = 2.12 ± 1.12°; *p* = 0.002; Pelvic obliquity GVS PwMS = 3.64 ± 0.93°; HC = 1.57 ± 0.59°; *p* < 0.001; independent-samples t-test), however, SPM found no differences in the frontal plane kinematics of such angles (Supplementary Figs. 2 and 3).

### Univariate analysis of kinetic data

**Figure 5:**
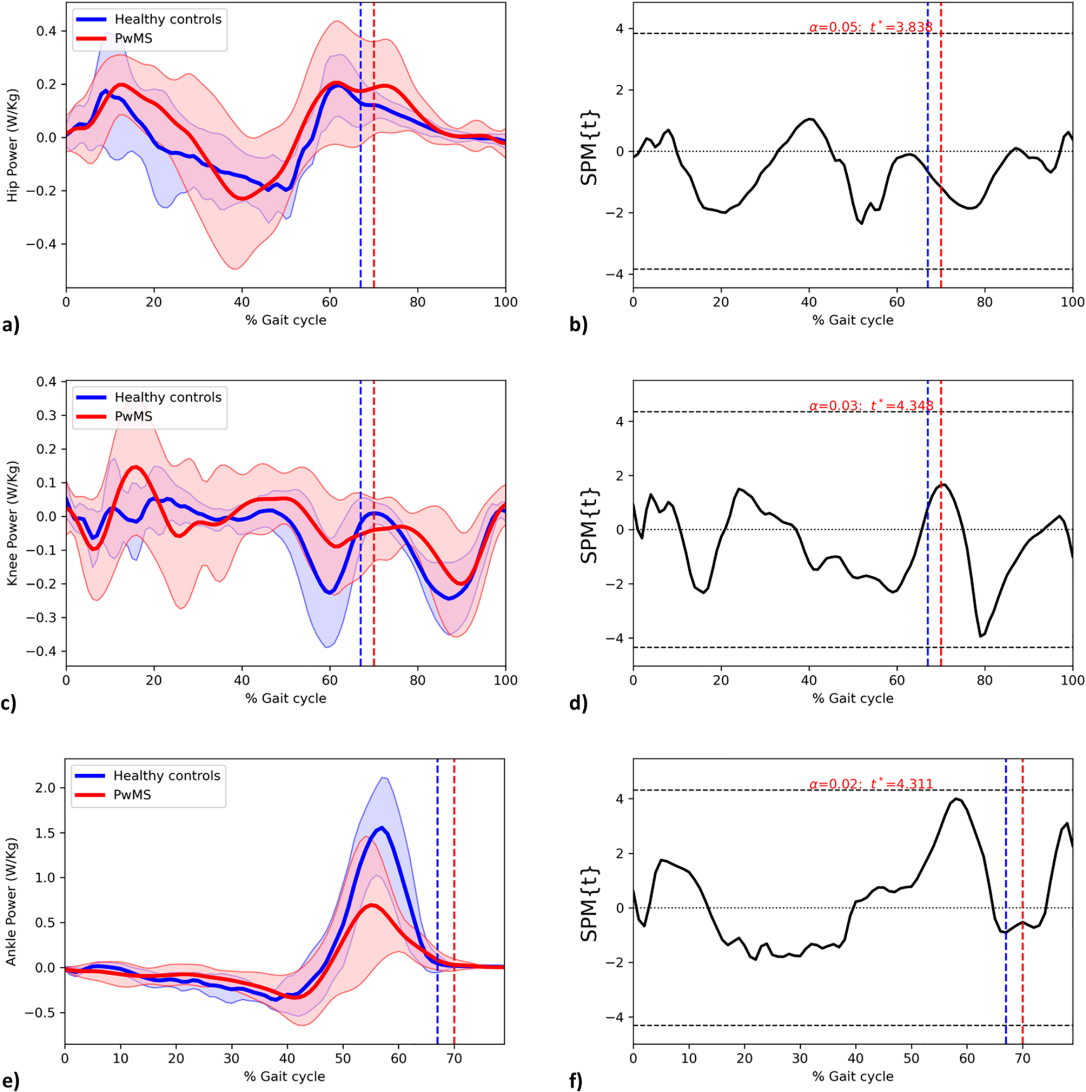
The hip, knee, and ankle power curves for PwMS and HC throughout the gait cycle. Mean hip (**a**), knee (**c**), and ankle (**e**) power as a function of the percentage of stride period in PwMS (red) and HC (blue), Standard Deviations of trajectories in PwMS (shaded red area) and HC (shaded blue area). Results of the independent-samples SPM t-test for hip (**b**), knee (**d**), and ankle (**f**) joints. *PwMS* People with multiple sclerosis; *HC* Healthy controls; *SPM* Statistical parametric mapping; Horizontal black dashed line, critical threshold for the two-sample SPM t-test; vertical dashed lines, mean foot–off in PwMS (red) and HC (blue).

An exploratory analysis of kinetic data was performed using two-sample SPM t-test comparing power at hip, knee, and ankle joints between PwMS and HC (Fig. 5). No statistically significant difference was present between the two groups, but a decreased ankle power was observed at around 55% of gait cycle in PwMS (Fig. 5).

### Correlations among GPS and clinical variables

Pearson’s correlation indexes between GPS and clinical outcomes were calculated. Mahalanobis distances were used to identify multivariate outliers, hence one participant was excluded from the correlation analyses. To display the influence of outlier removal, results of correlation analysis including the outlier are reported in Supplementary material. GPS correlated positively with disability level (EDSS: *ρ* = 0.65; 95%CI [0.04,0.91]; *p* = 0.04) and negatively with walking endurance (2MWT: *ρ* =  − 0.65; 95%CI [-0.91,-0.04]; *p* = 0.04), dynamic (DGI: *ρ* =  − 0.41; 95%CI [-0.83,0.30]; *p* = 0.24), and static balance (BBS: *ρ* =  − 0.25; 95%CI [− 0.78, 0.50]; *p* = 0.52).

## Discussion

This cross-sectional study aimed at identifying alterations in walking kinematics and kinetics in PwMS with moderate to severe disability, compared with speed-matched HC. These alterations were quantified through two different methods: GPS, a summary index of kinematics, and SPM, a statistical method that tests hypotheses over time-dependent kinematic and kinetic waveforms. PwMS had larger values of GPS (indicating greater deviation from normal physiological gait) than HC, with greater univariate deviations (i.e., GVS) at the knee and ankle joints on sagittal plane movements, while pelvis and hip kinematics were altered on frontal plane. Multivariate SPM on the sagittal plane showed that such deviations occurred mainly during the stance phase with kinematic alterations occurring mostly at the ankle and knee joints, probably due to a reduction of ankle power during the pre-swing phase.

### Multivariate differences between PwMS and HC

According to SPM Hotelling's T^2^ test, angular kinematics differed between PwMS and HC mainly during the stance phase (from loading response to terminal stance), and to a lesser extent during the swing phase (Fig. 1). Such multivariate analysis, which concertedly considers the hip, knee, and ankle kinematics, identified larger supra-threshold clusters both in number and size compared to univariate comparisons.

In the present study, alterations in multi-joint kinematics were confirmed by the higher GPS values in PwMS than in HC. We also observed higher GPS scores in PwMS than those previously reported by Pau et al. This can be explained by the higher disability level of our sample (EDSS: 5.5 ± 0.7) compared to the sample analysed by Pau et al. (EDSS: 3.5 ± 1.1)^10^.

Finally, the statistically significant correlations between GPS and disability level and walking endurance indicate that this index could be used to identify the walking motor impairment associated with disease severity.

### Univariate differences between PwMS and HC

Univariate analyses provided additional information about the individual contributions of the three joints.

Kinematic alterations of sagittal plane were mainly due to differences in ankle and knee joint kinematics, as highlighted by the higher GVSs of ankle and knee flexion in PwMS compared to HC. SPM revealed a cluster of reduced ankle dorsi-flexion in the stance phase (Fig. 2) that was coupled with reduced knee flexion during the heel-off (Fig. 3). Subsequently, during the swing phase alterations were mainly caused by reduced knee flexion while hip kinematics on sagittal plane seem less impaired across the whole gait cycle.

The reduced ankle dorsi-flexion in the stance phase has been widely observed in PwMS even at early stages of the disease^27^ Such reduced range of motion could be due to ankle joint stiffness, which has been hypothesised to be a functional protective mechanism to maintain balance whenever a deficit in postural control is perceived^27,28^.

Although SPM on kinetic data did not find statistically significant differences, a qualitative observation of kinetic curves highlights a reduced propulsive power generation in plantar-flexion activity among PwMS (Fig. 5e). This is in line with previous studies^29–32^ which found reduced maximal ankle power generation at toe-off during walking when compared with healthy controls. In line with these findings, Wagner et al. reported that ankle muscles weakness—but not spasticity—is a consistent predictor of walking dysfunction in PwMS, with plantar flexors giving a larger contribution than dorsiflexors^33^ We can hypothesise that the observed lack of power production at ankle level is a consequence of an insufficient eccentric plantar flexor activity during mid and terminal stance, which also limits the following heel rise and knee flexion, as confirmed by the cluster of statistically significant reduction in knee flexion during pre-swing in PwMS.

Along with alterations at the ankle, GVS also revealed a between-group difference in the knee Flexion–Extension. Besides alterations during the stance phase, SPM found a statistically significant difference in knee kinematics during the swing phase. The peak of knee flexion occurred approximately at 70% of the stride cycle during normal walking, while PwMS had a reduced and delayed knee flexion peak (occurring around 80% of gait cycle, Fig. 3a). In the present study, flexion limitation was also present from the initial part of swing phase of gait. Our results agree with previous studies reporting that impairment at peak knee flexion is a relevant feature of gait in PwMS and a valid predictor of walking function^34–36^ As suggested by the systematic review of Coca-Tapia et al., possible causes of this alteration are lower limb paresis, increase of muscle tone of knee flexors (included the rectus femoris), or decreased push-off power at the ankle joint^27^.

As previously mentioned, neither SPM nor GVS found statistically significant alterations in hip flexion–extension (Table 2; Fig. 4). This is in line with a previous study on PwMS with a low level of disability (EDSS < 4)^36^ but it is in contrast with a second paper showing limited extension and excessive hip flexion in PwMS with moderate to severe disability level^27^ Such discrepancy may be due to the influence of speed on hip kinematics: the first paper compared two speed-matched samples^36^ while the second considered results of PwMS and controls walking at different speeds^27^.

Though we hypothesized that sagittal kinematics would be more altered than the kinematics of the frontal plane, results of the GVS analysis highlighted significant differences in pelvic obliquity and hip abduction–adduction kinematics. This is in line with a recent review that showed increased frontal pelvic range of motion could be the results of compensatory strategies for altered distal movements during walking. To further investigate these frontal plane alterations, we performed SPM univariate comparisons that did not show any between-group differences in either segment (Supplementary Figs. 2 and 3). This lack of differences may be due to the large variability in pelvis kinematics, as depicted by the large red shaded area representing standard deviation of PwMS.

### Complementary use of GPS and SPM

GPS resulted suitable to quantify walking motor performance in PwMS, and its concurrent validity is supported by moderate correlations with validated clinical outcomes (r > 0.50)^37^ A further advantage of GPS is the possibility to be broken into 9 GVS components to identify what patterns deviate more from normal physiological patterns^9^.

Along with GPS, SPM provides valuable information on when during the gait cycle walking kinematics and kinetics diverge between PwMS and HC. Although other methods have been commonly used for this purpose—including the comparison of discrete (zero-dimensional) variables like peaks, means and phase durations^38^—SPM is preferable since it takes into account the waveform (one-dimensional) nature of these data^11,12^ When compared to zero-dimensional hypotheses tests, SPM allows to alleviate a priori assumptions about when statistically significant differences might occur and to consider inter-joint covariance in multi-joint analyses^11,12^.

Although giving coherent results, the use of GPS, GVS, and SPM should be seen as complementary. When clinicians are interested in comparing multi-joint kinematics between PwMS and HC, GPS can be used as a summary measure, while multivariate SPM allows testing hypotheses on kinematic and kinetic waveforms. In parallel, GVS and univariate SPM can be applied to single-joint comparisons.

### Clinical insights

The complementary use of GPS and SPM characterized the direction, magnitude, and timing of gait alterations in PwMS, providing a better understanding of subject-specific impairments.

Although a large between-subject variability was observed in this sample, we showed that PwMS more frequently had altered kinematics at the distal joints in the stance phase. In people with similar characteristics, tailored intervention could focus on eccentric plantar-flexion during the stance phase to facilitate the concentric burst of propulsive plantar flexor activity at push-off, improving both ankle and knee kinematics. Notably, this approach has already been used during rehabilitation resulting in an appropriate choice for gait rehabilitation^29,32^.

### Study limitations and future perspectives

This study comes with some strengths and limitations. Sample size was defined a priori, but the low number of participants did not allow to represent all the clinical heterogeneity of PwMS. These results can hence be generalized only to PwMS with similar moderate to severe level of disability, and further studies on PwMS with a wider range of disability level are needed to confirm the validity of the results.

To better investigate the impact of timing on kinematic parameters alterations, the use of temporal alignment techniques of gait events should be considered for future investigation. Moreover, electromyographic analyses are needed to compute muscle synergies, which are useful to model the complexity of motor control during gait and to investigate underlying neuromotor characteristics.

Finally, we did not collect data about participant’s cognitive function nor about lateral dominance making it impossible to assess their role in gait deviations.

## Conclusions

The present study described gait alterations in PwMS, introducing the complementary use of GPS and SPM. Our findings provide evidence that the largest gait alterations in PwMS involved the knee and ankle joints on sagittal plane and hip and pelvis alterations on frontal plane. Kinematic sagittal alterations during the stance phase were highlighted by both uni and multivariated analysis, the latter accounting for the movement of the three joints together. Although outside the aim of this study, the small sample provided initial evidence on the validity of the GPS that correlated positively with disability level and negatively with walking endurance.

## Supplementary Information

Supplementary Information.

## Data Availability

The datasets used and/or analysed during the current study are available from the corresponding author on reasonable request.
