# Supplementary material for: Complementary use of statistical parametric mapping and gait profile score to describe walking alterations in multiple sclerosis: a cross-sectional study
Source: Sci Rep. 2023 Jun 28;13:10465. doi: 10.1038/s41598-023-36916-5 (PMC10307864; doi:10.1038/s41598-023-36916-5)
Supplement: Supplementary file 1 — Supplementary Information. [file 41598_2023_36916_MOESM1_ESM.pdf]

Complementary use of Statistical Parametric Mapping and Gait Profile Score to describe walking alterations in multiple sclerosis: a cross-sectional study

Mestanza Mattos Fabiola Giovanna[1], Luciano Francesco[1], Lencioni Tiziana[2], Gervasoni Elisa[2]*, Jonsdottir Johanna[2], Anastasi Denise[2], Pavei Gaspare[1], Clerici Mario[1,2], Cattaneo Davide[1,2].

1 Department of Pathophysiology and Transplantation, Università degli Studi di Milano, Milan 20100, Italy.

2 IRCCS Fondazione Don Carlo Gnocchi, Milan 20148, Italy

*Corresponding author

Elisa Gervasoni, PhD

IRCSS Fondazione Don Carlo Gnocchi, Via Capecelatro 66 - 20148 Milan, Italy

email: egervasoni@dongnocchi.it., tel. +390240308814

Materials and methods

Gait profile score

Introduced by Baker et al., the Gait Profile Score (GPS) is a measure of the overall quality of gait kinematics that combines nine Gait Variable Scores (GVS): pelvic tilt, rotation and obliquity, hip flexion–extension, adduction–abduction and rotation, knee flexion–extension, ankle dorsiflexion and foot progression.[1] Each GVS was here calculated as the root mean square (RMS) difference between a patient's specific time-normalized gait variable and the average kinematic curve obtained from the healthy population across the gait cycle. Finally, the RMS average of all the 9 GVS will then equal the GPS.

Supplementary Table 1 and Supplementary Table 2 below explain how the kinematic variables used to calculate the nine GVS were calculated. Joint angles were calculated as the relative rotation between proximal and distal local reference frames, [2]which are defined by the direction of axes and planes determined by anatomical landmarks, associated to physical markers or calculated from them. [3]

Supplementary Table 1. Description of absolute and local reference frames used to determine the kinematic variables.

**Absolute reference frame**	**Description**
Functional frame	The vertical upwards Y axis coincides with the gravity line.<br>The forward X axis corresponds, in the gait trials, to the axis interpolating the posterior superior iliac spines (PSIS_MX) trajectory in the transverse plane (i.e. forward progression direction).<br>The Z axis is the cross-product between X and Y axes.
**Local reference frame**	**Description**
Pelvis	The forward-oriented X-axis passes through the midpoint between the posterior superior iliac spines (PSIS) and the midpoint between the anterior superior iliac spines (ASIS).<br>The upwards Y axis is perpendicular to the PSIS/ASISs plane.<br>The Z axis, pointing to the right, is the cross-product of X and Y axes.
Thigh	The thigh upwards longitudinal Y axis passes through the hip joint center (defined by the anthropometric measures) and the knee joint center (KJC, defined as midpoint between femur lateral condyle and femur medial condyle).<br>The forward X axis is perpendicular to the plane identified by the thigh longitudinal axis Y and by the vector defined by the femur condyles.<br>The thigh Z axis is the cross-product of X and Y axes.

Shank	The upwards longitudinal Y axis passes through the KJC and the ankle joint centre (AJC, midpoint between lateral and medial malleola).<br>The shank forward X axis is perpendicular to the plane identified by the shank longitudinal axis Y and by the vector defined by malleola.<br>The shank Z axis is the cross-product of X and Y axes.
Foot	The longitudinal X axis passes through the AJC and the midpoint between the first metatarsal head and fifth metatarsal head.<br>The upwards Y axis is perpendicular to the plane in the foot longitudinal axis X and the vector defined by metatarsal heads.<br>The Z axis is the cross product between X and Y axes.

Supplementary Table 2. Description of the nine kinematics variables used to calculate the Gait Profile Score.

**Kinematic variables**	**Description**
Pelvic angles	Reference frames: absolute and local pelvis.<br>The **pelvic tilt** is the pelvis rotation about the absolute transverse axis.<br>A floating axis is defined as the perpendicular axis to both the absolute transverse axis and the pelvic longitudinal axis. The **pelvis obliquity** is the angle about the floating axis.<br>The **pelvis rotation** is the angle about the longitudinal pelvis axis.
Hip angles	Reference frames: local pelvis and local thigh.<br>The **hip flexion** angle is the rotation about the pelvis transverse axis.<br>A floating axis is defined as the perpendicular axis to both the pelvis transverse axis and the thigh longitudinal axis. The **hip ab/adduction** is the angle about the floating axis.<br>The **hip rotation** is the angle about the longitudinal thigh axis.
Knee angles	Reference frames: local thigh and local shank.<br>The **knee flexion** angle is the rotation about the thigh transverse axis.
Ankle angles	Reference frames: local shank and local foot<br>The **ankle flexion angle** is the rotation about the shank transverse axis.
Foot angles	Reference frames: absolute and local foot<br>The **foot progression** (i.e., foot rotation) is the angle about the longitudinal foot axis.

Statistical Parametric Mapping and power analysis

In this paper, angular kinematic waveforms were compared between HC and PwMS using Statistical Parametric Mapping (SPM). Similar to common zero-dimensional (0D) tests, this procedure requires calculating a test statistics, a null hypothesis rejection threshold, and evaluating whether such threshold is exceeded by the test statistics. One-dimensional (1D) test statistics are defined by extending 0D ones over q time points. For a two-sample t-test (Pataky et al., 2013):

$$SPM\{t\} = \frac{\overline{y_B}(q) - \overline{y_A}(q)}{\sqrt{\frac{1}{J}\left(s_A^2(q) \; + \; s_B^2(q)\right)}}$$

where $\overline{y_A}$ and $\overline{y_B}$ are the sample means, s_a and s_b are the standard deviations, and J is the sample size. The original works from Pataky and colleagues[4–7] provide further details on how 1D test statistics and null hypothesis rejection thresholds are calculated.

A priori SPM power analysis was conducted to estimate the required sample size for this study.[6] This procedure requires to define a null (H0) and an alternative (H1) effect, make assumptions on the smoothness (NFWHM) and amplitude (NAMP) of the noise of the experimental data, and select a target false-positive and false-negative statistical error rate.[8–10] Power analysis was only done for hip, knee, and ankle kinematic comparisons as they were the primary endpoint of the study; all the other analyses should be deemed as exploratory. Previous research from Filli et al. compared walking kinematics between people with multiple sclerosis (PwMS; Age: 48.6 ± 10.3 years, height: 170.4 ± 9.9 cm, body mass: 68.5 ± 17.1 kg; Expanded Disability Status Scale: 4.5 ± 1.0) and healthy controls (HC; Age: 48.8 ± 10.1 years; height: 170.9 ± 7.0 cm; body mass: 71.7 ± 11.5 kg), providing useful information to define H0 and H1.[11] Data from this paper were hence digitized using WebPlotDigitizer v4.5 (https://automeris.io/WebPlotDigitizer) and interpolated over 101 points through a spline. For each angular comparison, the signal was calculated as the difference between the average waveforms of PwMS and HC groups. It was not possible to directly evaluate noise characteristics from Filli et al. as only mean waveforms and their standard deviations (SD) were published. However, the mean of the SD for each angular waveform did not relevantly differ between PwMS and HC; hence, NAMP in PwMS was assumed to be the same as in HC. According to the noise data on healthy participants from Luciano et al., we assumed a NAMP of 5°, 8° and 5° for hip, knee, and ankle joint kinematics, respectively, and a NFWHM of 30%.[9] Finally, power analysis was conducted for a SPM t-test comparing hip, knee, and ankle angular trajectories between two independent samples, with an allocation ratio of 1:1. Type II error was kept below 0.20 (i.e., statistical power higher than 0.8), while the family-wise error rate (α) was controlled through a Holm-Bonferroni correction in the form:

$$\alpha_i = \frac{\alpha}{(n - k + 1)}$$

where α was set to 0.05, n was the total number of multiple comparisons (3, in this case) and k was the significance rank for each comparison, which could be calculated by previously running a power analysis without multiple comparisons correction and sorting comparisons by their statistical power. The required sample sizes were 8, 7 and 11 per group for hip, knee, and ankle comparisons, respectively. Eleven participants per group were hence recruited to ensure an adequate *a priori* statistical power for all the three planned comparisons.

Supplementary Figure 1. *A priori* Statistical Parametric Mapping (SPM) power analysis for planned kinematic comparisons.

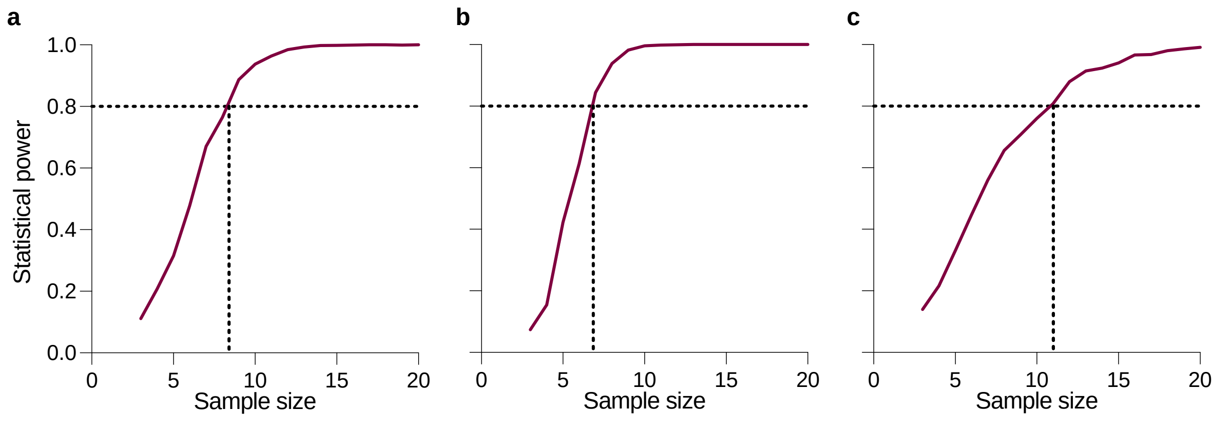

The relation between omnibus statistical power and the size of each sample is plotted for comparisons for hip (a), knee (b) and ankle (c) angular kinematics. The family-wise error rate was kept under 0.05 using the Holm-Bonferroni correction.

Results

Supplementary Figure 2. The Pelvis obliquity angle curves for PwMS and HC throughout the gait cycle.

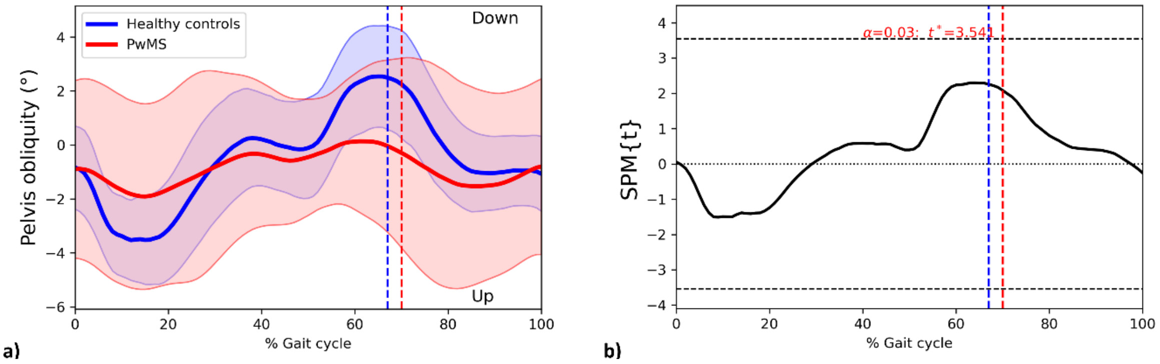

a) Mean trajectories for pelvic obliquity angles in PwMS (red) and HC (blue), Standard Deviations of trajectories for ankle dorsi- and plantar flexion angles in PwMS (shaded red area) and HC (shaded blue area). b) Results of independent samples SPM t-test. PwMS, People with Multiple Sclerosis; HC, Healthy Controls; SPM, Statistical Parametric Mapping; horizontal black dashed line, critical threshold for the two-sample SPM t-test; grey area, supra-threshold cluster; vertical dashed lines, mean foot-off in PwMS (red) and HC (blue).

Supplementary Figure 3. The hip adduction-abduction angle curves for PwMS and HC throughout the gait cycle.

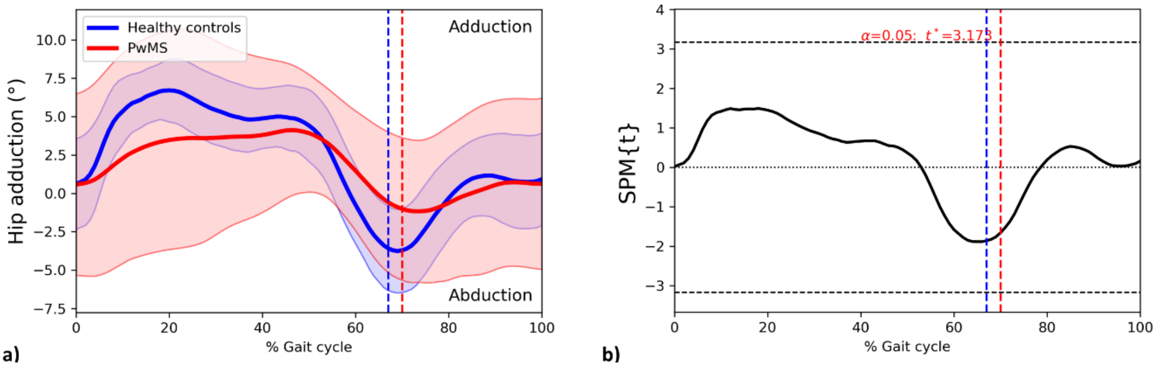

a) Mean trajectories for hip adduction-abduction angles in PwMS (red) and HC (blue), Standard Deviations of trajectories for ankle dorsi- and plantar flexion angles in PwMS (shaded red area) and HC (shaded blue area). b) Results of independent samples SPM t-test. PwMS, People with Multiple Sclerosis; HC, Healthy Controls; SPM, Statistical Parametric Mapping; horizontal black dashed line, critical threshold for the two-sample SPM t-test; grey area, supra-threshold cluster; vertical dashed lines, mean foot-off in PwMS (red) and HC (blue).

Supplementary Figure 4. Scatter plots of GPS and clinical outcome measures.

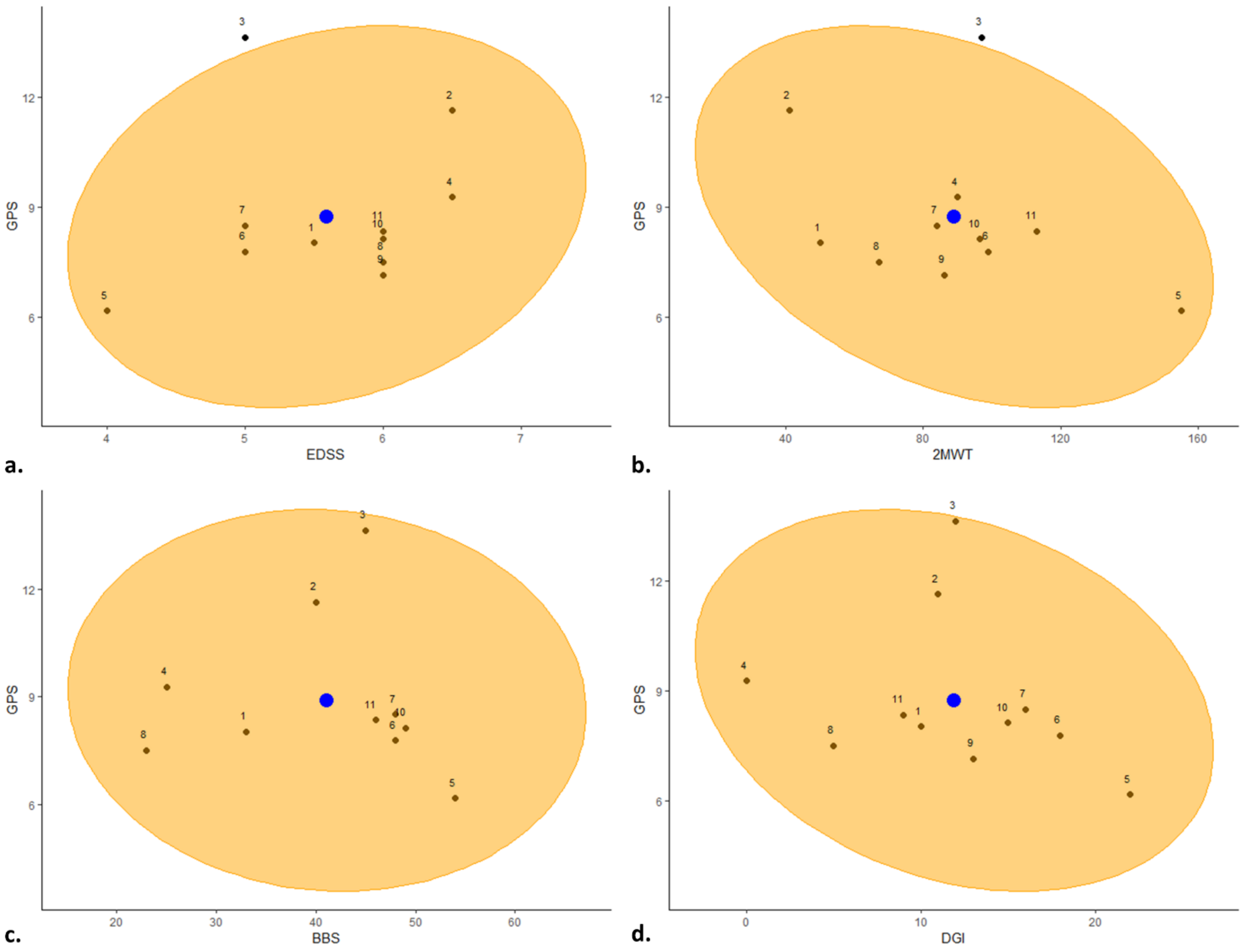

The yellow elliptic area contains cases considered non outliers based on Mahalanobis distance (95% CI).

GPS, Gait Profile Score; 2MWT, Two Minute Walking test; BBS, Berg Balance Scale; DGI, Dynamic Gait Index

Correlations between GPS and clinical variables

Pearson's correlation indexes (ρ) between GPS and clinical outcomes with the outlier case were: ρ=0.22 95%CI [-0.44, 0.72], p=0.52 with EDSS; ρ= -0.35 95%CI [-0.44, 0.72], p=0.29 2MWT; ρ=-0.06 95%CI [-0.67, 0.59], p=0.87 with BBS; and ρ=-0.26 95%CI [-0.75, 0.40], p=0.44 with DGI.

Supplementary references

1. Baker, R. *et al.* The Gait Profile Score and Movement Analysis Profile. *Gait Posture* **30**, 265–269 (2009).

2. Grood, E. S. & Suntay, W. J. A Joint Coordinate System for the Clinical Description of Three-Dimensional Motions: Application to the Knee. *J Biomech Eng* **105**, 136–144 (1983).

3. Rabuffetti, M. *et al.* The LAMB gait analysis protocol: Definition and experimental assessment of operator-related variability. *Proc Inst Mech Eng H* **233**, 342–353 (2019).

4. Pataky, T. C. Generalized n-dimensional biomechanical field analysis using statistical parametric mapping. *J Biomech* **43**, 1976–1982 (2010).

5. Pataky, T. C., Vanrenterghem, J. & Robinson, M. A. Zero- vs. one-dimensional, parametric vs. non-parametric, and confidence interval vs. hypothesis testing procedures in one-dimensional biomechanical trajectory analysis. *J Biomech* **48**, 1277–1285 (2015).

6. Pataky, T. C. Power1D: a Python toolbox for numerical power estimates in experiments involving one-dimensional continua. *PeerJ Comput Sci* **3**, e125 (2017).

7. Pataky, T. C., Robinson, M. A. & Vanrenterghem, J. Vector field statistical analysis of kinematic and force trajectories. *J Biomech* **46**, 2394–2401 (2013).

8. Robinson, M. A., Vanrenterghem, J. & Pataky, T. C. Sample size estimation for biomechanical waveforms: Current practice, recommendations and a comparison to discrete power analysis. *J Biomech* **122**, 110451 (2021).

9. Luciano, F., Ruggiero, L. & Pavei, G. Sample size estimation in locomotion kinematics and electromyography for statistical parametric mapping. *J Biomech* **122**, 110481 (2021).

10. Pataky, T. C., Robinson, M. A. & Vanrenterghem, J. A computational framework for estimating statistical power and planning hypothesis-driven experiments involving one-dimensional biomechanical continua. *J Biomech* **66**, 159–164 (2018).

11. Filli, L. *et al.* Profiling walking dysfunction in multiple sclerosis: characterisation, classification and progression over time. *Sci Rep* **8**, 4984 (2018).
